# Abnormal Cortical Development after Premature Birth Shown by Altered Allometric Scaling of Brain Growth

**DOI:** 10.1371/journal.pmed.0030265

**Published:** 2006-08-01

**Authors:** Olga Kapellou, Serena J Counsell, Nigel Kennea, Leigh Dyet, Nadeem Saeed, Jaroslav Stark, Elia Maalouf, Philip Duggan, Morenike Ajayi-Obe, Jo Hajnal, Joanna M Allsop, James Boardman, Mary A Rutherford, Frances Cowan, A. David Edwards

**Affiliations:** 1 Department of Paediatrics, Imperial College, Hammersmith Hospital, London, United Kingdom; 2 Division of Clinical Sciences, Imperial College, and MRC Clinical Sciences Centre, Hammersmith Hospital, London, United Kingdom; 3 Department of Mathematics, Imperial College, London, United Kingdom; Children's Hospital, United States of America

## Abstract

**Background:**

We postulated that during ontogenesis cortical surface area and cerebral volume are related by a scaling law whose exponent gives a quantitative measure of cortical development. We used this approach to investigate the hypothesis that premature termination of the intrauterine environment by preterm birth reduces cortical development in a dose-dependent manner, providing a neural substrate for functional impairment.

**Methods and Findings:**

We analyzed 274 magnetic resonance images that recorded brain growth from 23 to 48 wk of gestation in 113 extremely preterm infants born at 22 to 29 wk of gestation, 63 of whom underwent neurodevelopmental assessment at a median age of 2 y. Cortical surface area was related to cerebral volume by a scaling law with an exponent of 1.29 (95% confidence interval, 1.25–1.33), which was proportional to later neurodevelopmental impairment. Increasing prematurity and male gender were associated with a lower scaling exponent (*p* < 0.0001) independent of intrauterine or postnatal somatic growth.

**Conclusions:**

Human brain growth obeys an allometric scaling relation that is disrupted by preterm birth in a dose-dependent, sexually dimorphic fashion that directly parallels the incidence of neurodevelopmental impairments in preterm infants. This result focuses attention on brain growth and cortical development during the weeks following preterm delivery as a neural substrate for neurodevelopmental impairment after premature delivery.

## Abstract 2

Measurement of the way that the brain grows after birth in preterm infants, particularly the relation between surface area and cortical volume, may help to predict neurodevelopmental impairment.

## Editors' Summary

**Background.**

Nowadays, many children who are born prematurely can be expected to survive. However, children who are born very prematurely have a high chance of having brain damage that leads to delayed development compared with children of the same age but who were not born prematurely. This delay continues into school age at least and is worse in boys than in girls. Although some children have large obvious areas of brain damage, shown on brain scans, most do not and the changes that cause the delay must be more subtle. One possibility is that the underlying abnormality may be due to the fact that in these children the surface of the brain grows at too slow a rate compared to the volume of the brain. It is already known that the human brain (and the brain of closely related monkeys) has a very high surface area compared to its volume and that during normal development this surface area grows much faster than the volume. This extra growth appears to be necessary for the brain to make all the connections it needs.

**Why Was This Study Done?**

The researchers wanted to look at how the different parts of the brain grew in very premature babies born before 30 weeks (the normal time of gestation is around 40 weeks). In particular, they wanted to see if there were changes in the rates at which the different parts of the brain grew in relation to each other (the study of the change in proportion of various parts of an organism as a consequence of growth is known as Allometrics—hence the title of the paper). They then wanted to see if the rates of brain growth were affected by a variety of factors, including the sex of the baby or how premature he or she was, and whether there was a relationship between the rate of brain growth and later delayed development.

**What Did the Researchers Do and Find?**

Using a specially designed magnetic resonance imaging (MRI) machine—a type of scanner that allows very detailed pictures to be taken of the brain—the authors took 274 images that recorded brain growth from 23 to 48 wk of gestation in 113 extremely premature infants born between 22 and 29 weeks of gestation. 63 of these children were then assessed to see how they were developing mentally at around 2 years of age. As expected, the researchers found that the brain surface area grew faster than the brain volume and that the rate of growth was proportional to the chances of having delayed development later—that is, the slower the rate of growth of surface area relative to volume the more likely there was to be delayed development. In addition, the more premature babies, and those that were male, were most likely to have a slower growth of the brain surface compared with the brain volume.

**What Do These Findings Mean?**

It seems that human brains grow during development in a particular way, which means that the surface area grows more than the volume. When babies are born prematurely this pattern of growth is disrupted, and the amount of disruption of the growth seems to predict whether there is delayed development 2 years later. The earlier the birth, the greater the disruption is; in addition, boys are affected more than girls. These results will need to be confirmed in more babies, but if they are correct then it may be possible to monitor brain growth after birth in order to predict which children might need development support later on. The research also suggests useful avenues for further work to understand the exact neuroanatomy of disability in these children.

**Additional Information.**

Please access these Web sites via the online version of this summary at http://dx.doi.org/10.1371/journal.pmed.0030265.

• MedlinePlus has a page of links to information on premature babies

• The  March of Dimes is a US charity that funds research on prematurity

• Action Medical Research and Wellbeing are UK charities that fund research in this area

## Introduction

The evolution of large brains in primates required the solution of diverse geometrical problems, including the need for more and longer neural connections between increasingly distant cortical areas and the transport of substrates to meet the metabolic requirements of larger numbers of more distant cells. Recent computational work suggests that evolution has optimized these geometrical solutions in terms of microcircuit design, axon length, and areal adjacency [1–3] as well as material transport [4,5]. These characteristics are revealed in precise allometric scaling laws that reflect not only the geometric constraints but also the intrinsic functional properties of the neural system [6–8].

Cortical growth is achieved predominantly by an increase in surface area rather than thickness, and during late fetal human development a rapid increase in brain size occurs with exuberant development of cortical surface area relative to cerebral volume, manifested in the development of cortical convolutions. Many of the geometric and functional constraints that affect phylogenetic development must apply during ontogenesis, so we asked the simple question: Can brain growth during late human fetal development also be characterized by scaling laws; and more specifically, is growth of cortical surface area related to total cerebral volume by a power law? We reasoned that the scaling exponent would provide a sensitive measure of the development of the cortex, and that environmental pressures that reduce cortical growth would be detectable as a reduced scaling exponent. Analysis of scaling would then make it possible to discover specific factors that affect cortical development.

Brain development appears to be affected by premature termination of the intrauterine environment following preterm birth. Half of all surviving infants born at 25 weeks or less show neurodevelopmental impairment at 30 mo of age [9]. At age 6 y about 40% have cognitive impairment compared to their classroom peers, and impairments are more severe in boys than girls [10]. Even among less-immature infants over one third develop neurocognitive and behavioural problems [11–13]. This neurocognitive impairment is more severe with earlier gestation and longer exposure to the premature extrauterine environment [11,14], suggesting that there is a dose- and gender-dependent effect of prematurity on brain development.

No neural substrate has yet been described that accounts for this dose-dependent and sexually dimorphic effect of premature birth on cognitive function. It is not explained by large destructive brain lesions, which occur too infrequently to account for the high incidence of neurocognitive impairments [10]. Quantitative data on brain volumes have been reported [15,16], however, and evidence indicates that abnormal cortical development may be involved: Infants who survive prematurity appear to have reduced cortical surface area and less cortical grey matter compared to normal term-born infants, despite apparent preservation of total brain volume [17,18]; in late childhood imaging changes consistent with aberrant cortical development have been detected [19]; and localized abnormalities in specific cortical regions have been associated with particular cognitive impairments [20–23]. There are some longitudinal data in small groups of infants [24], but no previous study has systematically quantified cortical growth in a large, unselected cohort of preterm infants beginning immediately after premature birth. In investigating the effects of prematurity on the brain it is important to capture this vulnerable early period, and to study a large group of infants who accurately represent the preterm population.

We therefore examined the growth of cortical surface area relative to cerebral volume in a unique, unselected sequential cohort of extremely preterm infants serially imaged by magnetic resonance (MR) beginning soon after premature delivery until term-corrected age. We aimed to investigate the hypothesis that during this period growth in the cortical surface area and cerebral volume are related by an allometric power law whose scaling exponent is affected by the environmental pressure of prematurity in a dose-dependent, sexually dimorphic fashion, and is proportional to neurodevelopmental outcome.

## Methods

### Approval and Patient Consent

Ethical approval for MR imaging studies of preterm infants was given by the Hammersmith Hospitals Research Ethics Committee and written parental consent was obtained in each case.

### Patients

Between January 1997 and November 2000, all infants born at less than 30 weeks gestational age at the Hammersmith Hospital, London, were eligible for entry into a consecutive cohort study. The gestational age at birth was calculated from the date of the last menstrual period and confirmed with data from early antenatal ultrasound scans. In this time frame, 162 infants were born; median gestational age was 27 completed weeks (range 22–29 completed weeks) and median birth weight was 871 g (range 370–1,606 g). At birth, 37 infants (35.9%) were small for gestational age. All mothers received conventional antenatal corticosteroid therapy. Of the imaged infants, 22% were small for gestational age at birth, 42% had prolonged rupture of membranes, 73% were delivered by caesarean section, 48% had clinical evidence of chorioamnionitis, and 48% required additional oxygen at 36 wk gestation.

Consent was not available for 22 infants, but no significant difference was found in the birth weight or gestational age of these infants compared to those entered into the study. Of the final 140 infants enrolled in the study, 21 died before a scan could be performed; 119 infants were examined, of whom 106 survived to be discharged from the neonatal unit.

### MR Image Acquisition

We installed within our Neonatal Intensive Care Unit a unique 1.0 T MR imaging system (Oxford Magnet Technology, Oxford, UK) with full intensive care capacity that allowed serial imaging of sick infants from 23 wk gestational age onwards, and collected the database of serial images from the period immediately following delivery until term-corrected age. We previously published data on the safety and physiological parameters in this system [25]. Pulse oximetry, electrocardiographic, and visual monitoring were used throughout the examination, and each infant was attended by a neonatologist throughout the examination. Ear protection was used on each infant (Natus MiniMuffs, Natus Medical, San Carlos, California, United States). T2-weighted fast-spin echo images (TR = 3500 ms, TE_eff_ = 208 ms, echo train length = 16, matrix 192 × 256, field of view = 20 cm, slice thickness = 4 mm) acquired in the transverse plane were used for analysis.

We aimed to acquire serial images on infants from birth until term-corrected age, allowing for the practical constraints of neonatal intensive care. Infants underwent a median of two scans (range 1–8), and in total 327 image sets were obtained. All images were reviewed by a perinatal neuroradiologist, and infants with major destructive lesions (periventricular leucomalacia, hemorrhagic parenchymal infarction, post-hemorrhagic ventricular dilatation, and porencephalic cysts) were excluded from analysis. We found that 274 images from 113 infants of median gestational age at birth of 26.86 wk were free of major destructive lesions and of sufficient quality for computational measurement of cortical surface area and cerebral volume. The distribution of postnatal age of first scan was highly skewed: the mode was day 1, the median was day 2, and the interquartile range was day 1–4. In some infants imaging at term-corrected age was delayed by practical issues, and the dataset thus spans the period from 23 to 48 wk gestational age.

### Cortical Surface Area and Cerebral Volume Measurements

**Figure 1:**
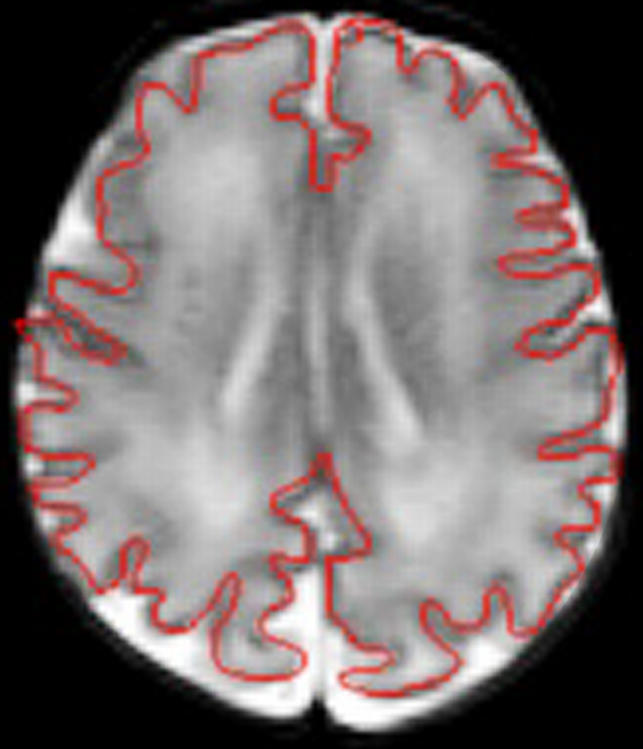
Annotated MR Image Showing Definition of Cortical Boundary

MR images analysed using a semiautomatic technique based on a method that was developed for use in adult brain analysis [26]. In brief, the boundary of the cortex was extracted semi-interactively using a combination of thresholding, manual tracing, and a contour-following algorithm (Figure 1). A contour consisting of line segments following the edges of voxels and marking the outer edge of the cortex as visualized on each slice was thus determined, and this was stored using the Freeman 8-way chain code [27]. The outer edge of the cortex could be seen clearly on each slice due to the high contrast between cortex and cerebrospinal fluid. Partial volume effects associated with the finite resolution of images necessarily made the precise definition of the cortical surface uncertain at the level of an individual voxel. However, this factor was common amongst all the patients analyzed.

Total cerebral volume (brain volume after exclusion of ventricular volume and the cerebellum) was calculated from the sum of the total number of voxels in the slices of brain assessed, less the number of voxels representing the ventricles and cerebellum multiplied by the planar dimensions of the voxels and the thickness of the brain slices. The surface area of the cortex was calculated from the total length of the measured cortex contour in each slice, defined as the number of voxel edges it contained, multiplied by the voxel dimension in plane and by the slice thickness, summed over all slices. Test-retest error was 2% for cerebral volume and 1% for cortical surface area.

### Cortical Surface Area: Cerebral Volume Scaling

If we measure the surface area *s* and volume *v* of a collection of three-dimensional objects of identical shape but different size we see a scaling relation of the form

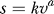
where *α* = 2/3 is a scaling exponent and *k* is a constant. Transforming to log coordinates we obtain

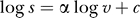
where *c* = log *k*. Thus if we plot log *s* against log *v* the data will lie on a straight line of slope 2/3. In the case of a developing brain, we expect the surface to become relatively larger as the brain grows, and hence it is plausible to postulate a scaling relationship but with a larger value of *α*. This value can be estimated by plotting a scatter graph of log *s* against log *v* and determining whether the data lie approximately on a straight line. If so, the slope determined using linear regression gives an estimate of *α*.

Our hypothesis was that premature birth would lead to a reduced rate of growth of cortical surface area. In terms of the above model this effect would appear as a significant dependence of the exponent *α* on the gestational age *g* at birth (in particular, we expect *α* to increase with *g*). The simplest form of such dependence is linear, so that *α* is replaced by the term *α*_0_ + *α*_1_ *g.* It is also necessary to allow *c* to vary with *g*, so that the regression model becomes


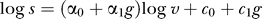


To determine whether the scaling exponent or the effect of gestational age were dependent on gender *(d);* intrauterine growth *(b);* or postnatal growth *(w),* with growth determined as body weight expressed as a z-score to account for measurements made at different gestational ages [28], an extended model was developed:


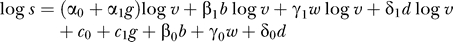


The model was fitted using regression analysis. *α*_1_ *g* was determined by the interaction term (cerebral volume × gestation) and the effects of growth and gender by analogous interactions. We employed cross-sectional time series analysis by generalized least-square regression for random effects using the *xtreg* command in STATA 8.0 software (Statacorp, http://www.Stata.com). This method provided a robust variance estimate for deriving confidence intervals (CIs) and for hypothesis testing, which accounted for variable number and timing of measurements in individual participants. Similar results were obtained using standard regression analysis with robust estimation of standard errors to allow for non-independence of data within individuals. To visualise an intuitive measure of the dependence of scaling on gestational age at birth we calculated and displayed the scaling exponent for infants born in a particular gestational birth week.

To explore the nature of this scaling relation further we examined the rate of growth of both the volume and the surface area components over time. Using an analogous generalized least-squares regression for random effects approach we estimated the effect of gestational age at birth, gender, intrauterine growth, and postnatal growth at the time of scan on the rate of change of cerebral volume and cortical surface area over time.

### Neurodevelopmental Examination

Neurodevelopment was assessed at median 23.83 mo (5–95 centiles, 19.5–28.9) corrected age by experienced neuropaediatricians unaware of the MR imaging data. A full Griffiths Mental Development Scales: Revised 1996 (The Test Agency, Henley-on-Thames, UK) assessment was performed, and developmental quotients (DQs) were calculated for each subscale (termed locomotor, personal & social, hearing & speech, eye & hand coordination, and performance) and for the total score. The standardized mean (± standard deviation) total score for the term born population was 100 (± 12) and for the subscales it was 100 (± 16). Complete neurodevelopmental data were available for 63 infants.

The relationship between neurodevelopmental outcome and gestational age at birth was examined by estimating the linear regression; because the age of examination was not identical in all infants, the corrected age at examination was included in the model to test for systematic bias. We made further exploratory analyses for each neurodevelopmental subscale.

## Results

**Figure 2:**
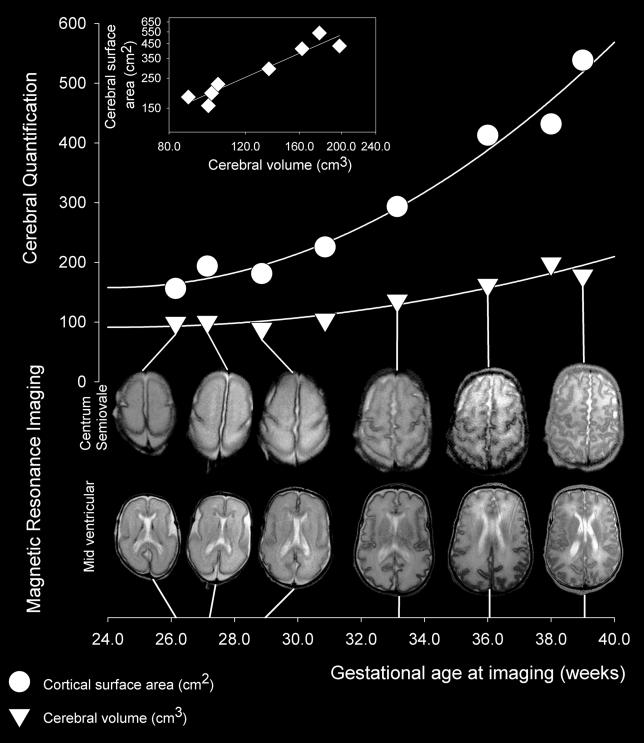
Serial MR Imaging of Brain Growth in a Normal Female Preterm Infant When this infant was born at 25 weeks gestational age she weighed 710 g. The images show slices through the brain at the mid-ventricular level and at the level of the centrum semiovale from six of the eight MR images obtained between 26 and 39 wk gestational age; images obtained at 30 and 38 weeks are omitted for graphical clarity. Measured values for cerebral volume (triangles) and cortical surface area (circles) are related to relevant image pairs by straight lines. The insert displays a scatter plot in log-log coordinates of cortical surface area and cerebral volume (diamonds), showing a linear relationship that indicates power law scaling of cortical surface area relative to cerebral volume in this individual.

Data from a “normal” female infant born at 25 wk gestational age, weighing 710 g, whose DQ was 106 are shown in Figure 2. The first image was obtained three days after delivery. Birth weight was normal, but postnatal growth was reduced, with body weight at 39 wk gestational age 2.86 standard deviations below expected. Paired image slices at the level of the centrum semiovale and mid-lateral ventricle obtained between 26 and 39 wk gestational age are shown together with measured values for cerebral volume and cortical surface area. The scatter plot of cortical surface area and cerebral volume in log-log coordinates for this individual shows a linear relationship with a value of 1.42 (95% CI, 0.98–1.87), which indicates power law scaling.

**Figure 3:**
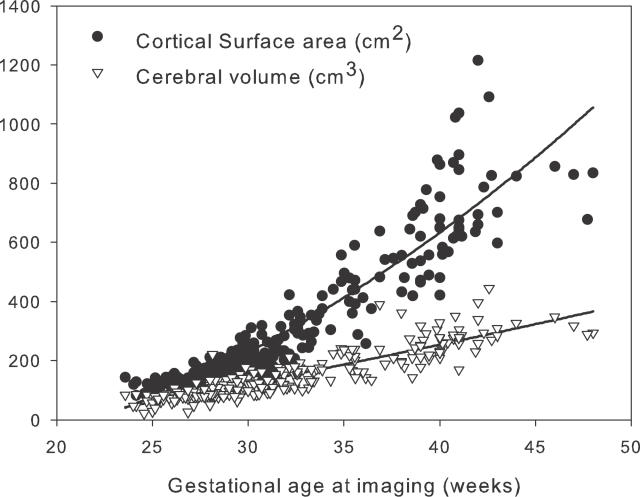
Cortical Surface Area and Cerebral Volume of Preterm Infants A total of 113 preterm infants, born between 23 and 30 weeks gestation, were measured for cortical surface area and cerebral volume over the period from birth to 48 weeks gestational age.

**Figure 4:**
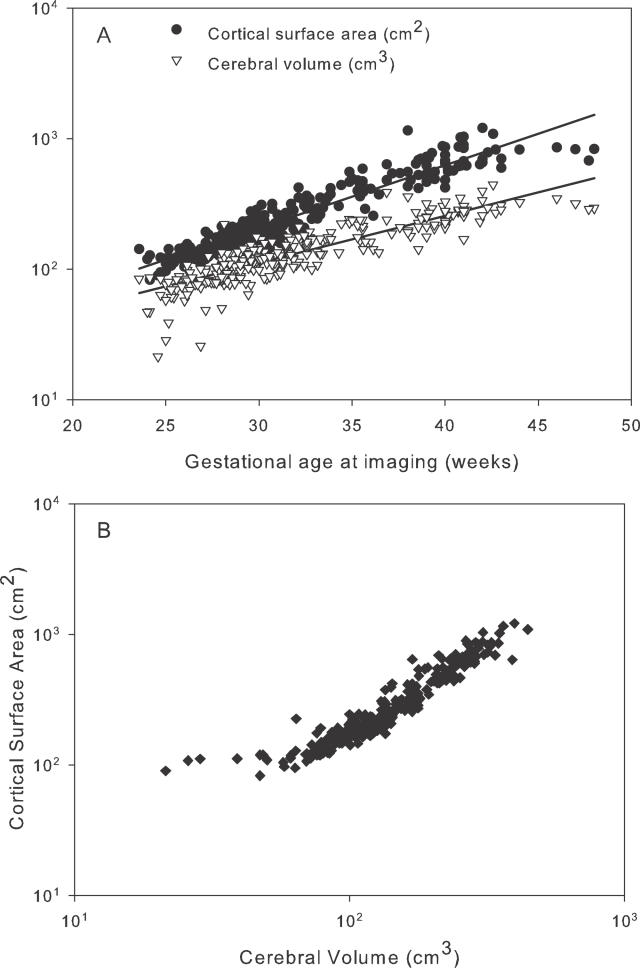
Cerebral Volume and Surface Area Growth in Preterm Infants (A) Values for cerebral volume and cortical surface area for 113 infants imaged serially between 23 and 48 wk gestation (274 scans) displayed on log-log coordinates and related to gestational age at scan, showing linear relationships for both variables. (B) Scaling of cortical surface area relative to cerebral volume shown as a scatter plot in log-log coordinates of the cortical surface area *s* against cerebral volume *v* determined from each of these 274 scans. The linear relationship apparent for volumes greater than about 45 cm^3^ indicates a power law scaling of the form *s* = *kv^α^*, where *α* is the scaling exponent and *k* is a constant. For a three-dimensional object that maintains its shape with changing size, the exponent *α* would take the value 2/3 (since area scales as length squared and volume as length cubed). For the data in this plot, in contrast, a good fit (for *v* > 45 cm^3^) is obtained with *α* = 1.29 (95% confidence interval 1.25–1.33).

Measured values for cerebral volume and cortical surface area from all participants are given in Figure 3. Figure 4 shows that the log-transformed values approximated to a straight line consistent with a power law relation (*p* < 0.0001). Four influential outliers were defined at the lower extreme of the distribution with cerebral volume values of less than 45 cm^3^; when these outliers were removed, the scaling exponent *α* was 1.29 (95% CI, 1.25–1.33); inclusion of the outliers produced a measured scaling exponent of 1.18 (95% CI, 1.14–1.23).

The extended model of the effect on the scaling exponent of gestational age at birth together with the potential influences of growth and gender showed that the *α*_1_ was positive (0.096 [95% CI, 0.069–0.124]), indicating that the surface area to cerebral volume scaling exponent increases significantly with gestational age at birth (*p* < 0.0001). Neither intrauterine nor postnatal growth accounted for significant variance, however a small but significant reduction existed in male compared to female brain growth (*p <* 0.001). The regression results for the expanded model are given in Table 1. Inspection of added variable plots showed good fits and homogenous variance.

**Table 1:**
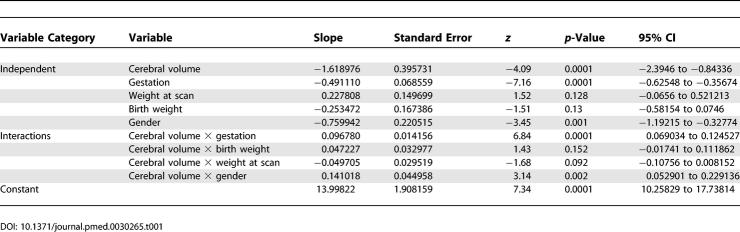
Regression Analysis of the Extended Model Using a Generalised Least Squares Random Effects Approach

Exploration of the individual components of the scaling relation showed that the growth in cerebral volume was not related to any variable, while the growth in cortical surface area was significantly related to gestational age at birth (0.004 cm^2^ wk^−2^ [95% CI, 0.001–0.007]) but not gender, birth weight, or body weight at the time of scan. The sexually dimorphic effect of prematurity on the scaling exponent therefore could not be explained simply by a change in one component. However, further exploration of the interaction between prematurity and gender was not possible, because data were insufficient to fit the more complex model.

**Figure 5:**
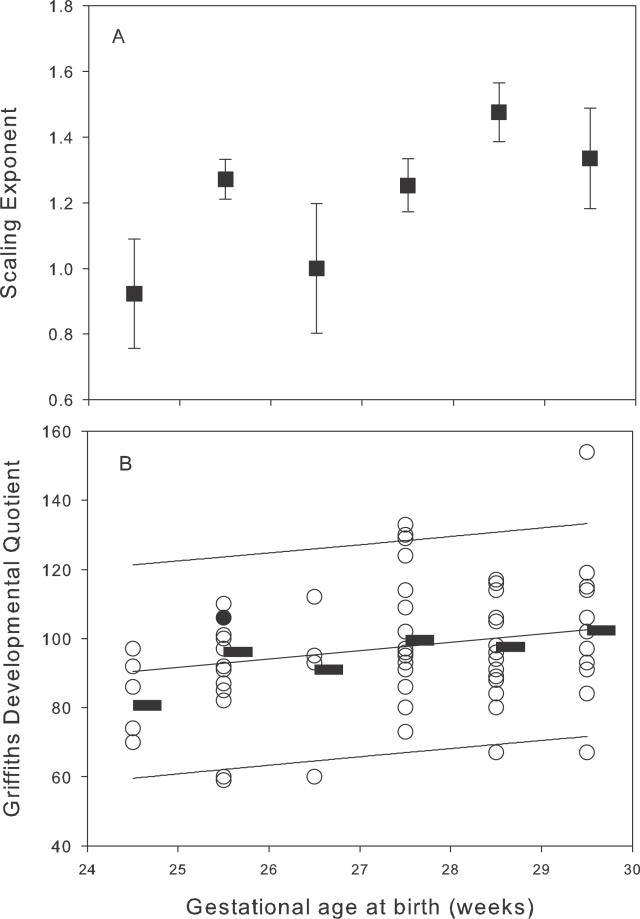
Effect of Increasing Prematurity on Brain Growth and Neurodevelopmental Function (A) Scaling exponent *α* determined for each birth week cohort (mean and 95% confidence intervals for the mean), showing decreased values at earlier gestations, giving an intuitive univariate visualization of the generalized least-squares random effects modelling. (B) Neurodevelopmental outcome for infants born in these weeks, showing both the DQ for each individual assessment (hollow circles) and the mean value for each birth week (horizontal bar) with regression line and 95% confidence limits for the population. It can be seen to increase with gestational age at birth (*p <* 0.05). The single filled circle denotes the infant presented in Figure 2.

Neurodevelopmental data were available for only three infants born at less than 24 wk gestational age, so these infants were analysed together with infants born at 24 wk gestation. The DQ was positively related to gestational age at birth (*p* = 0.008), and there was no significant effect due to the age of neurological examination. Figure 5 displays the positive relation of DQ to gestational age at birth; included in the plot to aid visualization is the scaling exponent *α* calculated for infants born in each birth week. Exploratory analysis of the relation between the component subscales of the neurodevelopmental score and the scaling exponent showed a positive univariate relation between scaling exponent and all subscales except the locomotor.

## Discussion

This study demonstrated that human brain growth obeys a gender-specific allometric scaling law during development, and that the scaling exponent is sensitive to environmental effects: Premature loss of the intrauterine environment due to preterm birth led to a dose-dependent reduction in the scaling exponent; this directly parallels the incidence of neurodevelopmental impairment characteristic of extremely preterm infants. The effects of gender and prematurity were independent, but although we suspect an increased susceptibility of boys to the effects of prematurity, our dataset was insufficient to demonstrate this effect formally.

The measurement of surface area and cerebral volume by our semi-interactive approach is conceptually and practically simple, avoids technical problems inherent in measurements of cortical volumes and thickness [29], and captures the most significant morphological changes that occur during this period. However, the imaging methods used have limitations. The approach does not render the cortex as a continuous smooth surface but realizes it as a series of small steps in which the vertical faces of the steps compose the calculated surface area. Other approaches to surface estimation would produce slightly different absolute values, although the imprecision would be small given that the surface area being measured is large on the scale of voxels, and a slice thickness of 4 mm might cause some underestimation of more complex surface anatomies. However, any underestimation would reduce rather than increase the chance of observing a significant effect of prematurity on the scaling exponent, and thus does not undermine the conclusions of this study.

Although this study demonstrates the first application, to our knowledge, of allometric analysis to human brain development in the context of preterm delivery, the approach has been used repeatedly to investigate the increase in brain size during mammalian evolution [6,8,30]. Estimation of scaling exponents is attractive because it provides a dimensionless metric of brain development that is immediately understandable in physical and mathematical terms and that allows large datasets to be combined and compared. The calculation of scaling exponents in evolutionary studies requires caution due to problems with allometric grades and phylogenetic inertia [31]. These are not significant features of the current developmental dataset; however, an additional characteristic problem—influential outliers at the extremes of the distribution—was identified. These outliers were dealt with by removing four data points, a small number compared to the size of the whole dataset (274); as a result, the value of the scaling exponent was altered slightly, but no substantive change affected the analysis.

The surface area and volume of a three-dimensional object of invariant shape scales by an exponent of 2/3. Many biological systems display a pervasive scaling of 1/4 power, particularly in the relation between body mass and metabolic or circulatory parameters. It has been argued that this scaling relates to the central importance of material transport and reflects the fractal geometry of branching systems [4], or to other general properties of distributed transport networks [5].

In the nervous system, evolutionary scaling laws appear to encapsulate essential features of neural organization. Some reflect the solution of brain-specific problems and do not conform to the common 1/4 power scaling. For example, cortical grey matter scales to white matter by an exponent of 4/3, which theoretical analysis suggests is due to the constraints on brain cytoarchitecture induced by the predominance and nature of association fibres in subcortical white matter, with a disproportionably increasing volume required for communication fibres as the cortex grows in size [8]. The number of neurons in the lateral geniculate nucleus scales over evolution to the number of neurons in the primary visual cortex by 3/2 power, a property that in the visual system appears to be due to the necessary properties of visual representation of a two-dimensional retinal space in a three-dimensional cortical space [6].

Brain growth during ontogeny appears to be subject to geometrical constraints similar to those of phylogenetic growth. Increasing relative cortical size and connectivity requires longer corticocortical connections, increased mass of connecting fibres, and functional connectivity that has similar implications for areal relations. Whole-body ontogenic growth has been usefully described by scaling relations: West et al. [32] have derived a general growth equation for a wide range of species from the basic principals of conservation of energy, allometric scaling of metabolic rates, and energy costs. They show that allometric measures can capture ontogeny, which is an important principle because evolutionary scaling in the brain is often predicated on measurements and assumptions that may not always be valid for ontogenic growth; for example, during evolution, synapse density appears to be a fundamental invariant [33], and in apes and primates it is often assumed (not always correctly) that neuronal number per unit of surface area is relatively invariant [34]. Yet during the period of human development under investigation synapse density is changing, with a rapid increase followed by a decline in the postnatal period, and neural number, at least in some parts of the cortex, declines rapidly [35].

The allometric analysis in this study has uncovered precise quantitative information on brain development. Increases in cortical surface area relative to cerebral volume due to more rapid cortical growth is reflected in a scaling exponent higher than the 2/3 seen in an expanding object of invariant shape. The measured global exponent of 1.29 quantifies the disproportionate growth of cortex relative to cerebral volume; this approximately 4/3 scaling may reflect constraints induced by white matter growth analogous to those seen over evolution [8], although caution is required in this interpretation, as the numerical interpretation of scaling exponents is susceptible to small errors in measurement [31]. Further investigation would be possible using segmentation of MR images to separate and measure grey and white matter. Segmentation is difficult in extremely preterm infants, particularly in the separation of deep grey and white matter. Future improvements in segmentation algorithms can be expected that will facilitate further investigation [36].

Lower values of the scaling exponent in more preterm infants demonstrate reduced relative cortical growth in these infants compared to those with shorter exposure to the extrauterine environment. The observed effect of prematurity on the scaling exponent was large: In evolutionary allometry, much smaller differences in scaling exponent have precipitated major and sometimes controversial reinterpretations [37]. The effect was independent of intrauterine and postnatal body growth and was not due to major destructive brain lesions, although gender had a significant effect on this relation, which may relate to data suggesting that boys may be more vulnerable to brain injury and neurological impairment after preterm birth [38,39]. The growth in cerebral volume and cortical surface area did not show any dependence on gender; thus, although the effect of gestation on the growth rate of cortical surface area was quantitatively small, these components cannot fully explain the observed effect on allometric growth.

The dosage effect of premature birth on cortical development revealed by examination of the scaling exponent is, to our knowledge, the first clear example of aberrant neural development that parallels the well-described dosage effect of prematurity and the influence of gender on later neural function [11,14]. In addition, the close relation between prematurity, cortical growth, and neurodevelopmental functioning suggests that aberrant cortical development may be a neural substrate for abnormal function in preterm infants. Our dataset excluded the uncommon major destructive brain lesions that are closely related to motor deficits and cerebral palsy, and it may be noteworthy that in the exploratory analysis of the neurodevelopmental subscales, all except the locomotor were directly proportional to cortical growth, suggesting that aberrant cortical development is related to neurocognitive rather than neuromotor impairment. Others have identified morphologic cerebral alterations in association with neurologic impairment in later childhood and adolescence following preterm birth [20,21,40–44], but this literature is limited as it contains no analysis of dosage effect nor of how the effects of preterm birth may be modified by later events.

We looked for confounding influences and considered whether sampling could have produced an artificial result deriving from the fact that no images were obtained before delivery and not all infants were examined over the same time period. However, the change in shape from preterm to term brain is greatest in the most preterm, and thus the sampling artefact should have induced a greater scaling exponent in these infants, while we observed the opposite. It is also mathematically possible that the change in scaling might be due to increased growth in cerebral volume in the most preterm infants; however, our measurements of total cerebral volume revealed no difference between preterm and control infants [17]. We also ruled out intrauterine or postnatal whole-body growth as explanations of the observed effect.

We also considered whether MR imaging might have led to bias—particularly whether using images with the relatively large slice thickness of 4 mm could cause a systematic inaccuracy in surface area measurement. However, any bias induced by the thick image slices would cause underestimation of more complex anatomies, so that the less preterm infants with more complex brains might appear to have smaller cortical surface areas than the true value; this could have led to an underestimation of the effect of prematurity but does not affect the robustness of the conclusions. In addition, the measurement of surface area rather than other measures of cortical growth such as cortical thickness or cortical grey matter volume avoids problems identified in some of these measurements [29].

It seems likely that reduced growth of the cerebral cortex is due to reduced connectivity rather than a reduced number of cortical neurons. The radial unit model of cortical neurogenesis, for which there is strong experimental support [45], states that neocortical area is dependent on the number of cortical founder neurons in the ventricular zone during early fetal life and is reduced by increased premitotic apoptosis very early in development; however, this stage is complete before viable preterm birth, and increased neuronal death later in development reduces cortical thickness rather than cortical area [45]. Evolutionary allometry suggests that cortical scaling is dependent on growth of association fibres rather than change in cortical cell number [8], and one attractive interpretation of the present allometric analysis is that it records abnormal corticocortical connectivity as a substrate for impairment. Association fibres and synaptic development play an important role in fine-tuning cortical growth during late fetal and early extrauterine life, and this may be disrupted by prematurity. In rodents, for example, damage to connectivity in the cortical subplate, a prominent human structure at 23–32 wk gestational age, disrupts cortical development [46,47].

This allometric analysis establishes cortical development as vulnerable to the multiple immunological, nutritional, pharmacological, and other stresses of premature exposure to the extrauterine environment [48], and as a neuroanatomic correlate for later cognitive impairment. The study emphasises, however, that prematurity does not damage the cerebral cortex in isolation, but disrupts the coordinated growth of the whole brain. This implies that understanding the mechanisms of neurological impairment in preterm infants will require not the study of focal lesions or isolated structures, but a “systems approach.” Neuroimaging and neuroinformatics now offer tools that may help achieve this, particularly if underpinned by a theoretical framework based on the development of neural organization.

## Abbreviations

CI: confidence interval
DQ: developmental quotient
MR: magnetic resonance
